# ASSOCIATION BETWEEN PAIN AND FALL WORRY AMONG COMMUNITY-DWELLING OLDER PEOPLE WITH DEMENTIA IN THE UNITED STATES

**DOI:** 10.1093/geroni/igad104.0781

**Published:** 2023-12-21

**Authors:** Yuanjin Zhou, Tatiana Sadak, Nayanika Ghosh, Elizabeth Phelan

**Affiliations:** University of Texas at Austin, Austin, Texas, United States; University of Washington, Seattle, Washington, United States; University of Texas at Austin, Austin, Texas, United States; University Of Washington, School of Medicine, Seattle, Washington, United States

## Abstract

Previous studies have found that pain is associated with fall worry among community-dwelling older adults. However, both pain and fall worry are poorly understood and under-addressed among community-dwelling older people with dementia (cd-OPWD). We thus sought to examine the association between pain and fall worry among this vulnerable subgroup. We used data from the 2015 National Health and Aging Trends Study (analytic sample: cd-OPWD who were self-interviewed, n=1103; mean age: 79; age range: 65-107). The number of pain sites in the prior month was assessed by presenting the cd-OPWD with a card listing common pain sites (e.g., back, knees). Past-month fall worry was assessed by two questions, “did you worry about falling down” and “did this worry ever limit your activities”. Fall worry was endorsed by 34%; activity-limiting fall worry was endorsed by 39% of those with fall worry. Twenty percent reported a single site of pain, and 53% reported multisite pain. Those who endorsed fall worry were more likely to have a higher number of pain sites, and those who reported activity-limiting fall worry were more likely to report knee, hand, or leg pain. In multinomial logistic regression analyses, adjusting for sociodemographic and health characteristics, a higher number of pain sites was significantly associated with activity-limiting fall worry. These findings suggest pain and fall worry are common among cd-OPWD and can be elicited directly from them. Fall prevention for cd-OPWD should prioritize pain management to mitigate activity-limiting fall worry, since activity limitation increases the risk of falls.

